# SNAP‐Tag–Based Recombinant Photoimmunotherapeutic Agents for the Selective Detection and Killing of Light‐Accessible Melanotransferrin‐Expressing Melanoma and Triple‐Negative Breast Cancer

**DOI:** 10.1002/cam4.70912

**Published:** 2025-05-06

**Authors:** Suzanne Hippolite Magagoum, Fleury Augustin Nsole Biteghe, Gael Tchokomeni Siwe, Dirk Lang, Nkhasi Lekena, Stefan Barth

**Affiliations:** ^1^ Medical Biotechnology and Immunotherapy Research Unit, Institute of Infectious Disease and Molecular Medicine (IDM) University of Cape Town Cape Town South Africa; ^2^ Department of Chemistry and Chemical Biology, College of Science Northeastern University Boston Massachusetts USA; ^3^ Division of Physiological Sciences, Department of Human Biology, Faculty of Health Sciences University of Cape Town Cape Town South Africa; ^4^ Department of Integrative Biomedical Sciences, Faculty of Health Sciences, South African Research Chair in Cancer Biotechnology University of Cape Town Cape Town South Africa

**Keywords:** melanoma, melanotransferrin, NIR‐PIT, single‐chain antibody fragment (scFv), SNAP‐tag–based antibody fusion protein, triple negative breast cancer

## Abstract

**Background:**

Melanoma and triple negative breast cancer (TNBC) represent the most aggressive skin and breast cancer subtypes and are associated with poor diagnostic and limited therapeutic options leading to poor prognosis. Melanotransferrin/p97 (MTf), initially identified as a tumor‐associated antigen (TAA) in melanoma, is overexpressed in various solid tumors, including TNBC. Beyond its high differential expression and dreadful tumorigenic impact, MTf is also associated with chemoresistance development, and its inhibition significantly hampers tumor progression, making MTf a promising target for effective targeted therapies. Near‐infrared photoimmunotherapy (NIR‐PIT) is an approach that combines the precision of antibodies directed against specific TAA with the phototoxic effects of a light‐sensitive photosensitizer (IR700), activated by near‐infrared (NIR) light irradiation. This study aimed to generate a novel photoimmunoconjugate to specifically destroy MTf‐positive melanoma and TNBC cells in vitro following NIR light irradiation.

**Methods:**

A single‐chain variable fragment (scFv) assembled from anti‐MTf antibody L49 was recombinantly fused with the SNAP‐tag protein (L49(scFv)‐SNAP), capable of irreversible and autocatalytic conjugation to any O(6)‐benzylguanine (BG) substrate in a 1:1 stoichiometry. Purified full‐length SNAP‐tag–based fusion protein (L49(scFv)‐SNAP‐tag) was either conjugated to a BG‐modified fluorescent imaging agent (Alexa 488) to specifically assess its selective binding to MTf‐expressing cell lines via confocal imaging and flow cytometry or to a BG‐modified light‐sensitive photosensitizer (IR700) to evaluate its phototoxic properties using an XTT cell viability assay.

**Results:**

The selective binding and internalization of L49(scFv)‐SNAP‐Alexa 488 towards MTf‐positive melanoma and TNBC cell lines were successfully demonstrated with MTF expression percentages ranging from 52.8 to 83.1. Once confirmed, dose‐dependent phototoxicity of L49(scFv)‐SNAP‐IR700 was achieved on illuminated MTf‐positive cell lines showing IC_50_ values in the nanomolar range (2.20–5.24 nM).

**Conclusion:**

This study highlights the therapeutic potential of MTf as a promising target for the diagnosis as well as selective and efficient elimination of NIR‐light‐accessible melanoma and TNBC by NIR‐PIT.

**Trial Registration:**

NCT03769506

**Abbreviations**
ABC: ATP‐binding cassette
ADC: antibody‐drug conjugate
APC: antibody‐photoabsorber conjugates
BG: benzylguanine
DAR: drug‐to‐antibody ratio
ER: estrogen receptor
GPI: glycophosphatidylinositol
HER2: human epidermal growth factor receptor 2
ICD: immunogenic cell death
IMAC: immobilized metal affinity chromatography
mAbs: monoclonal antibodies
MTf/p97: melanotransferrin
NIR: near‐infrared
PDT: photodynamic therapy
PIT: photoimmunotherapy
PR: progesterone receptor
PS: photosensitizers
ROS: reactive oxygen species
scFv: single‐chain variable fragment
SP: side population
TAA: tumor‐associated antigen
TNBC: triple negative breast cancer

## 1 Introduction

Melanoma and triple negative breast cancer (TNBC) are both the most aggressive types of skin and breast cancers, respectively, and are associated with a very poor prognosis [1, 2, 3, 4]. Despite their initial effectiveness, the current systemic therapies for advanced melanoma (chemotherapy, radiotherapy, targeted chemotherapy) are highly resistant and toxic to normal cells [5, 6, 7, 8, 9]. On the other hand, the inherent heterogeneity of TNBC along with the lack of tangible molecular targets (estrogen receptor [ER], progesterone receptor [PR], and human epidermal growth factor receptor 2 [HER2] gene) represents challenging obstacles to its effective treatment [10, 11]. Therefore, both tumors are also associated with limited therapeutic options.

Melanotransferrin/p97 (MTf), first identified as a melanoma‐associated antigen, is a 97 kDa iron (Fe)‐binding member of the transferrin superfamily [4, 12, 13]. Studies using monoclonal antibodies (mAbs) raised against melanoma cells reported that MTf was found on about 90% of allogeneic melanomas, versus about 55% of other tumors tested [4, 13], with a low differential expression in human adults' normal tissues [14, 15]. MTf was reported to have two forms: the glycophosphatidylinositol (GPI)‐anchor form bound to the plasma membrane (mMTf) and its short soluble version shed into serum (sMTf), which lacks the C‐terminal GPI anchor and can cross the blood–brain barrier [16, 17, 18, 19]. MTf is functionally related to human serum transferrin members such as transferrin and lactotransferrin because it binds Fe [19, 20, 21] and is involved in a variety of physiological processes including Fe metabolism and cellular differentiation [18, 22]. Further studies recently reported significant expression of MTf on a variety of solid tumors such as squamous non‐small cell lung cancer (NSCLC), colorectal, pancreatic, mesothelioma, and TNBC [15, 18, 23]. MTf expression directly affects the function of genes involved in membrane transport, endothelial cell migration and angiogenesis, cell proliferation, invasion and survival, plasminogen activation, and differentiation [19, 23, 24]. Additionally, downregulation of MTf was correlated with decreased tumor cell proliferation and significant reduction of tumor growth in nude mice melanoma, thus demonstrating a role of MTf in proliferation and tumorigenesis [4, 22, 24]. Moreover, delayed tumor growth associated with reduced development of human melanoma brain metastasis was reported in a mice model after single administration of an MTf‐targeting antibody, suggesting that this antigen plays a role in malignant tumor proliferation and migration [4, 25, 26]. Importantly, recent studies further confirmed that MTf was less expressed in normal tissues except brain endothelium, skin epidermis, sweat and salivary gland ducts and kidney tubules [15, 27, 28], indicating that the MTf antigen is a promising target for diagnosis of solid tumors and their targeted treatment.

Besides the ATP‐binding cassette (ABC) transporters effect, which is one of the common mechanisms of chemoresistance shared by various cancers [29, 30, 31], melanoma cells display specific features: (i) the presence of side population (SP) characterized by their efflux capacity of anticancer drugs from tumors [32, 33]; (ii) melanoma cells are equipped with melanogenesis‐related vesicles named melanosomes that are reported to be involved in drug trapping and export [6, 34]; (iii) resistance in melanoma was also partly attributed to a resistant cancer stem‐like cells population endowed with high clonogenic potential [35]. Additionally, stable transfection of MTf, followed by its overexpression, positively influences the expression of several genes such as ABCB5 transporter [24]. Therefore, a therapeutic regimen that selectively kills cancer cells while activating the local host immune response and escaping the effect of melanosomes, SP, and MTf would be ideal for effectively treating MTf‐positive solid tumors, for instance, melanoma and TNBC. One such approach could be near‐infrared photoimmunotherapy (NIR‐PIT) [36].

Conventional photodynamic therapy (PDT) emerged as one of the promising cancer treatment regimens, and it involves the use of tumor‐localized photosensitizers (PS) [37]. During PDT, PS can passively accumulate into tumors and produce death‐inducing amounts of reactive oxygen species (ROS) post‐light exposure in the presence of molecular oxygen, thereby causing tumor cell phototoxicity, vascular damage, and initiation of acute local and systemic inflammation [38, 39, 40]. However, the therapeutic efficacy of PDT can be limited by: (1) suboptimal tumor tissue penetration of therapeutic light and (2) passive diffusion of PS in normal tissues, which might lead to clinically undesired side effects [41, 42, 43]. To mitigate these unwanted effects and increase the specificity of PDT, antibody‐photoabsorber conjugates (APCs) consisting of light‐sensitive PSs chemically conjugated to tumor‐specific monoclonal antibodies (mAb) or their derivatives were developed for photoimmunotherapeutic application (PIT) [42, 44]. NIR‐PIT is a newly developed PIT that makes use of monoclonal antibody antitumor‐specific antigen conjugated to near‐infrared photoabsorber dye like IRDye700DX (IR‐700) with a high therapeutic absorbance wavelength in the range of NIR light [36, 45, 46]. After the binding of an mAb‐IR700 conjugate to the specific tumor antigen and upon NIR‐light irradiation, targeted cells rapidly undergo necrosis, which induces immunogenic cell death (ICD) in a highly selective manner, as evidenced by real‐time microscopy demonstrating swelling, bursting, and blebbing of the target cell membrane within minutes of light irradiation [47, 48, 49]. Another advantage of NIR‐PIT is that the PS IR700 is a hydrophilic photo‐dye with no phototoxic or bio‐toxic properties on its own. Therefore, unbound IR700 that dissociates from the APC is safe and, in addition, rapidly excreted in urine 24 h after NIR irradiation [46].

Traditional methods of antibody conjugation give rise to heterogeneous antibody‐drug conjugate (ADC) products with drug‐to‐antibody ratios (DAR) ranging from 0 to 8 because of random chemical conjugation resulting in inconsistent pharmacokinetic, efficacy, and safety profiles [50, 51]. SNAP‐tag is a modified version of the human DNA repair enzyme alkylguanine‐DNA alkyltransferase able to specifically and rapidly react with O(6)‐benzylguanine (BG)–modified substrates through an irreversible transfer of an alkyl group to a cysteine residue within its active site [52]. The unique antibody format created by fusing SNAP‐tag to a recombinant antibody fragment therefore provides a simple, controlled, and robust site‐specific method for self‐labeling of antibodies with different synthetic effector molecules [53, 55, 56] with a 1:1 stoichiometry [52, 54].

In this study, we generated recombinant fusion protein by genetically fusing SNAP‐tag to MTf‐specific single‐chain variable fragments derived from antibody L49 for optical imaging and NIR‐PIT against MTf‐expressing melanoma and TNBC cell lines.

## 2 Materials and Methods

### 2.1 Cell Lines

Human embryonic kidney cell line HEK‐293T (ATCC: CRL‐11268) and melanoma cell line SK‐MEL 28 (ATCC: HTB‐72) were cultured using Roswell Park Memorial Institute (RPMI‐1640) medium (containing 2 mM L‐glutamine, 3.7 g/L NaHCO_3_ and 15 mg/L phenol red, USA). Melanoma cell line A2058 (ATCC: CRL‐11147) and adenocarcinoma breast cancer cell lines MDA‐MB 231 (ATCC: HTB‐26) and MDA‐MB 468 (ATCC: HTB‐132) were cultured in Dulbecco's modified Eagle medium (DMEM). Media were supplemented with 10% (v/v) heat‐inactivated and gamma‐irradiated fetal bovine serum (FBS) and 1% (v/v) penicillin–streptomycin (100 U/mL). Media and additives were purchased from Gibco by Life Technologies (RPMI‐1640: Gibco #10566 and DMEM: Gibco #61870), supplied by Thermo Fisher Scientific, South Africa. The cells were maintained at 37°C in a 5% CO_2_ incubator with 95% humidity.

### 2.2 Engineering of Recombinant Plasmid DNA and Expression of SNAP‐Tag–Based Fusion Protein

The gene sequences of variable heavy (VH) and light (VL) chains of the humanized anti‐melanotransferrin antibody L49 were extracted from the US WO 98/50432 patent [57] and were subsequently subjected to IgBLAST analysis as described previously [54]. Upon confirmation of the presence of intact complementarity‐determining regions (CDRs) and framework regions (FRs), both chains were joined into a scFv format using a short glycine‐serine linker [54], and the newly designed scFv was synthesized at GenScript (GenScript; USA). Through a series of restriction enzyme digestions and ligation, the scFv was inserted into the mammalian expression vector pCB‐SNAP pre‐digested by *SfiI* and *NotI* to generate a mammalian expression vector with an open reading frame bearing an N‐terminal 6x histidine‐tag (6xhis‐tag), followed by the MTf targeting L49 scFv genetically fused to the C‐terminal SNAP‐tag. Successful cloning was confirmed by Sanger sequencing.

The generated plasmid DNA was transfected into HEK‐293T cells using the lipid‐based transfection reagent X‐tremeGENE HP (Sigma‐Aldrich, South Africa). Enrichment of transfected cells expressing enhanced green fluorescent protein (eGFP) was performed under Zeocin selection (150–200 μg/mL) until reaching a 90%–100% confluency. eGFP expression was employed to monitor efficient transfection through ZOE Fluorescent Cell Imager (Bio‐Rad Laboratories, UK). Expressed SNAP‐tag–based recombinant fusion protein was secreted into the cell culture supernatant (CCSN) as previously described [10, 54]. Using the 6xhis‐tag N‐terminal region, the recombinant fusion protein was purified from harvested CCSN through an immobilized metal affinity chromatography (IMAC) purification process using a 5 mL Ni^2+^ Sepharose affinity column (His‐Trap Excel, GE Healthcare, USA) mounted on an ÄKTA *Avant* 25 system (GE Healthcare, Germany), as previously reported [10, 54].

### 2.3 Sodium Dodecyl Sulfate–Polyacrylamide Gel Electrophoresis and Western Blot Analysis

Produced recombinant SNAP‐tag–based fusion protein was detected by sodium dodecyl sulfate–polyacrylamide gel electrophoresis (SDS–PAGE) and western blot as previously reported [10, 54]. Briefly, 15 μg of protein sample added to 4× Laemmli sample buffer (Bio‐Rad, USA) in a 3:1 ratio v/v and heated at 98°C for 5 min were run on two 10% SDS–PAGE gels against prestained protein ladder (Thermo Scientific) for 1 h 30 min at 120 V. The first gel was stained (1 h) using AcquaStain solution (Vacutec, South Africa) and the protein was visualized based on its molecular weight. SDS–PAGE image was captured using Gel Doc XR System (Bio‐Rad, USA). The N‐terminal 6xhis‐tag of the fusion protein was detected by western blot analysis. To this end, protein from a second, unstained SDS–PAGE gel was transferred to a polyvinylidene fluoride (PVDF) membrane as previously described [10, 54]. The membrane was immediately blocked with fat‐free milk (1 h) at room temperature and was subsequently incubated with a mouse anti‐polyhistidine−horseradish peroxidase (HRP) antibody (A7058‐1VL, Sigma Aldrich, USA) (1/2000 dilution in fat‐free milk) for 2 h. After three successive washing steps with 10 mL TBST buffer, the membrane was blotted with 2 mL of Pierce 1‐Step Ultra TMB‐Blotting Solution (1–2 min) (Thermo Scientific, USA) for colorimetric detection of L49(scFv)‐SNAP.

### 2.4 Site‐Specific Conjugation of SNAP‐Tag–Based Fusion Protein to BG‐Modified Substrates

#### 2.4.1 Conjugation of SNAP‐Tag–Based Fusion Protein to BG‐Modified Alexa Fluor 488

Produced full‐length fusion protein (5 μM) was incubated with BG‐modified Alexa Fluor 488 (10 μM) (New England Biolabs, USA) at a 1:2 M ratio for 30 min in the dark at room temperature, in 1× PBS supplemented with 1 mM dithiothreitol (DTT) (Sigma‐Aldrich, South Africa). Alexa 488–labeled fusion protein was run on a discontinuous 10% SDS–PAGE gel, and the fluorescent signal was visualized using an iBright FL1000 Imaging System (Invitrogen, Thermo Fisher Scientific, South Africa).

#### 2.4.2 Conjugation of SNAP‐Tag–Based Fusion Protein to BG‐PEG_24_ ‐IR700

Lyophilized BG‐PEG_24_‐IR700 kindly gifted by Professor Matthias Peipp (University of Kiel, Germany) was solubilized in 50% (v/v) DMSO (Sigma‐Aldrich, South Africa) and incubated with the produced full‐length fusion protein in a 3:1 M ratio for 3 h in the dark at room temperature. Unconjugated excess dye was removed using 10 kDa molecular weight cut‐off (MWCO) sized Amicon filters (Sigma‐Aldrich, South Africa) following the manufacturer's instructions. Successful generation of APC or IR700‐labeled fusion protein was assessed by running the conjugated product on a discontinuous 10% SDS–PAGE gel before visualization through a red‐fluorescent signal detection using an iBright FL1000 Imaging System (Invitrogen, Thermo Fisher Scientific, South Africa).

### 2.5 Binding Studies

The PS IR700 has superior imaging properties for in vivo optical imaging, and traditional in vitro imaging devices (confocal microscopes and flow cytometers available at UCT) are unable to detect narrow near‐infrared fluorescence peak [58]. Therefore, the fluorescent imaging agent Alexa 488 was used instead to assess the binding capacity of the antibody moiety of the produced recombinant fusion protein in vitro.

#### 2.5.1 Confocal Microscopy

Cell surface binding and internalization of Alexa Fluor 488–labeled fusion protein on melanoma and TNBC cell lines were assessed using confocal microscopy. Briefly, melanoma cell lines (A2058 and SK‐MEL‐28), TNBC cell lines (MDA‐MB‐231 and MDA‐MB‐468), and HEK‐293T cell line were seeded to a density of 5 × 10^4^ cells per well in 4 quadrants CELLview cell culture dishes, glass bottom (Greiner bio‐one, Germany) (live cell imaging) or on a coverslip in a 35‐mm dish (for fixed cells imaging) and incubated for 48 h at 37°C. Afterward, cells were washed twice with 1× PBS and incubated for 30–60 min with 20 μg of Alexa 488–labeled fusion protein in 200 μL of serum‐free medium (0.2 μg/μL). Thereafter, cells were washed with 1× PBS and incubated with 200 μL of 1:5000 dilution Hoechst nuclear counterstain (Thermo Fisher Scientific, South Africa) for 5 min. Excess dye was removed by washing the cells three times with 1× PBS and 200 μL of serum‐free media was added to the cells before imaging. For fixed cell imaging, after washing out excess dye, cells were fixed with 4% (v/v) paraformaldehyde (PFA) (Sigma‐Aldrich, South Africa) for 20 min at room temperature and then washed with 1× PBS. The coverslip was afterward mounted on a microscope slide (using Mowiol mounting medium from Merck, USA, and anti‐fade). The slides were left to dry in the dark at 4° overnight. Images were acquired using the Zeiss confocal scanner microscope (LSM 880, USA) with Airy Scan (Confocal and Light Microscope Imaging Facility, University of Cape Town, South Africa) on the 65× and 40× air objectives, respectively, for live and fixed cell imaging.

#### 2.5.2 Flow Cytometry

The quantitative binding of Alexa Fluor 488–labeled fusion protein on MTf‐expressing cell lines was assessed by flow cytometry. Beforehand, the maximal amount of fluorophore‐labeled probe allowing a clear separation between MTf‐positive and MTf‐negative cell lines was determined to forestall any overlay of background signal that can be revealed by flow cytometry [59, 60]. To this end, different concentrations of Alexa Fluor 488–labeled fusion protein (0.01, 0.1, 0.2, 1, and 2 μg/μL) were incubated with MTf‐negative cells HEK293T for flow cytometry analysis to determine which concentration has the least overlay of background while allowing a clear separation between negative and positive populations. Subsequently, 5 × 10^5^ cells were incubated wi th LIVE/DEADᵀᴹ (L/D marker) Fixable Violet Dead Cell Stain (Thermo Fisher Scientific, South Africa) (1/1000) at room temperature for 30 min and were washed with 1× PBS. Afterward, cells were incubated with Alexa Fluor 488–labeled‐fusion protein (with the chosen concentration) on ice for 60 min and were washed three times using 1× PBS. Data were acquired using a BD LSRFortessa Cell Analyzer (30,000 events) (BD Biosciences, USA) and analyzed using FlowJo software (v10.8.1) (BD Biosciences, USA).

### 2.6 Cell Viability Assay

Photocytotoxicity of generated APC after activation by near‐infrared light was assessed using the XTT cell proliferation assay, and the whole experiment was carried out in the dark. Cells (5 × 10^3^) were seeded in 96‐well plates in triplicate and incubated for 30 h in a 5% CO_2_ atmosphere with 95% humidity. The next day, cells were incubated with fivefold serially diluted concentrations of APC and incubated for 3 h at 37°C untreated cells, treated cells, and positive control treated with Zeocin (400 μg/mL). Concurrently, untreated cells were subjected to optimal NIR light to assess the possible heat‐related phototoxicity. After washing with 1× PBS, the cells were maintained in 200 μL of supplemented phenol red‐free media (RPMI‐1640: Gibco #11835‐030 or DMEM: Gibco #21063‐029) prior to irradiation with optimal NIR‐light wavelength (690 nm, 17 mW/cm^2^, 40 J/cm^2^) [61] for 13 min [58]. Irradiated cells were washed twice with 1× PBS and incubated for 30 h in supplemented medium under standard cell culture conditions to allow them to recover. Cell viability was assessed using a cell proliferation kit II (XTT) (Roche, Switzerland) according to the manufacturer instructions. Briefly, 50 μL of XTT reagent mixed with its electron coupling reagent, in a 1:0.02 M ratio, was added to cells and incubated for 4 h under standard cell culture conditions. Afterward, cell viability was determined by colorimetric monitoring of XTT reduction to formazan at 450 nm absorbance wavelength and 655 nm emission wavelength using the iMark Microplate Absorbance Reader (Bio‐Rad, USA). Each experiment was carried out in triplicate (*n* = 3) and was performed in three technical replicates. Data were normalized with respect to the negative (100% cell viability) and positive (0% cell viability) controls, and the results were presented as percentages of cell viability against the log of drug concentration. The concentration corresponding to a 50% reduction in cell viability (IC_50_ value) was determined using GraphPad Prism v10.1.1 software (https://www.graphpad.com/).

### 2.7 Statistical Analysis

Dose‐dependent cytotoxicity absorbance values were analyzed using GraphPad Prism v10.1.1 software. One‐way ANOVA followed by Mann Whitney *U*‐test was used to assess the significant difference between untreated cells exposed to NIR‐light and their corresponding controls. *p* < 0.05 was set to determine statistical significance. Unless otherwise specified, data were expressed as mean ± standard error of the mean (SEM).

## 3 Results

### 3.1 Molecular Cloning, Expression and Characterization of L49(scFv)‐SNAP

The anti‐MTf L49(scFv) antibody gene sequence (extracted from US patent WO 98/50432) was successfully genetically modified through in silico cloning by joining the VH and VL chains using a short glycine‐serine (G_4_S)_3_ linker sequence and by inserting unique restriction enzyme cutting sites *Sfi1* and *Not1*. Upon synthesizing the newly designed L49(scFv) (by GenScript; USA), it was successfully inserted into a mammalian expressing plasmid (pCB‐) bearing a SNAP‐tag DNA sequence to generate the recombinant pCB‐L49(scFv)‐SNAP plasmid via a series of restriction enzyme digestions and ligation (data not shown).

**FIGURE 1:**
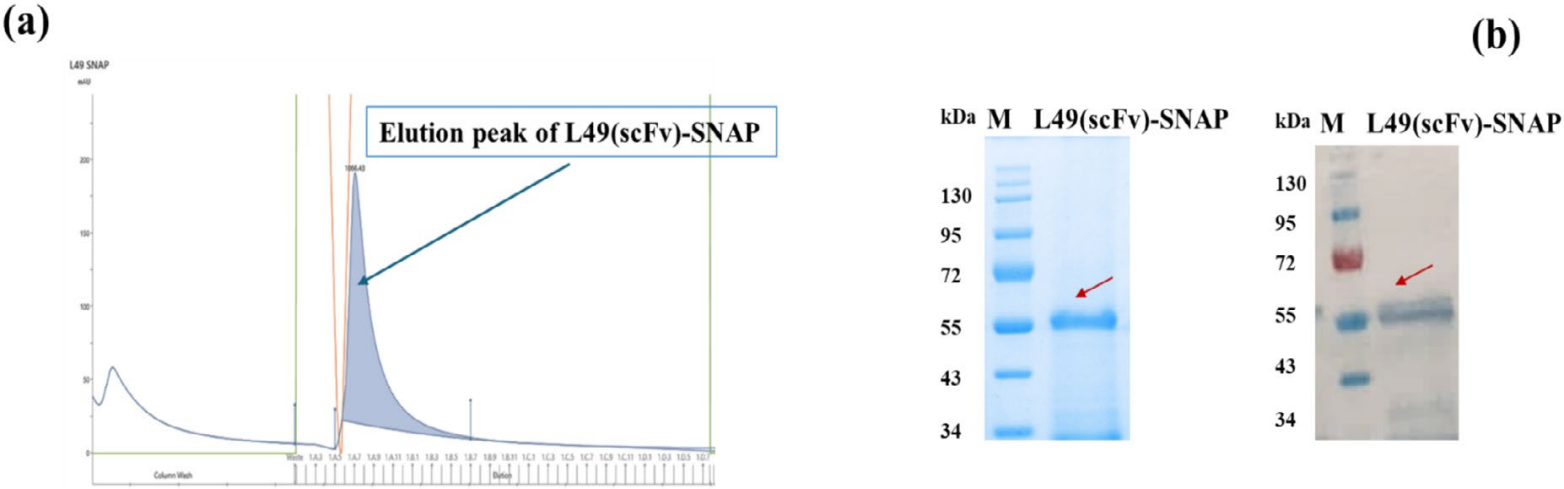
Purification and characterization of L49(scFv)‐SNAP. (a) IMAC elution profile of L49(scFv)‐SNAP. The *y*‐axis indicates absorbance in milli absorbance unit (mAU), and the *x*‐axis represents the ÄKTA flow‐through volume over time. The green line represents imidazole concentration and indicates that elution of L49(scFv)‐SNAP was achieved within a single peak as displayed on the chromatogram (filled blue curve). (b) Characterization of purified L49(scFv)‐SNAP. The first SDS–PAGE gel (left‐hand side) was stained using AcquaStain solution and the fusion protein was identified according to its theoretical protein size (51.4 kDa). The second gel was transferred to the PVDF membrane and following incubation with anti‐polyhistidine−HRP antibody, L49(scFv)‐SNAP was visualized after the addition of blotting buffer. The SDS–PAGE gel image was taken using Gel Doc XR System (Bio‐Rad, USA). L49(scFv)‐SNAP is indicated on both stained SDS–PAGE gel and WB membrane by red arrows. kDa, kilo dalton; M, protein standard; scFv, single chain variable fragment.

Recombinant fusion protein L49(scFv)‐SNAP‐tag was successfully expressed transiently in HEK293T cells. The recombinant protein released into the CCSN was collected and purified by IMAC. Full‐length L49(scFv)‐SNAP bound to the Ni^2+^ affinity column during sample application, via its his‐tag N‐terminus, was eluted in a single‐step elution peak following the application of a high concentration of imidazole (250 mM) (Figure 1a) after extensive washing. Elution fractions were concentrated and concomitantly run on two 10% SDS–PAGE gels to specifically detect L49(scFv)‐SNAP based on its theoretical size (51.4 kDa). The stained gel revealed the presence of enriched recombinant protein L49(scFv)‐SNAP at the expected theoretical size (Figure 1b, left‐hand side). Western blot analysis, performed using the second unstained gel, confirmed the presence of the 6xhis‐tag‐bearing L49(scFv)‐SNAP on the PVDF membrane at the expected theoretical size (Figure 1b, right‐hand side).

### 3.2 Site‐Specific Conjugation of L49(scFv)‐SNAP to BG‐Modified Substrates

**FIGURE 2:**
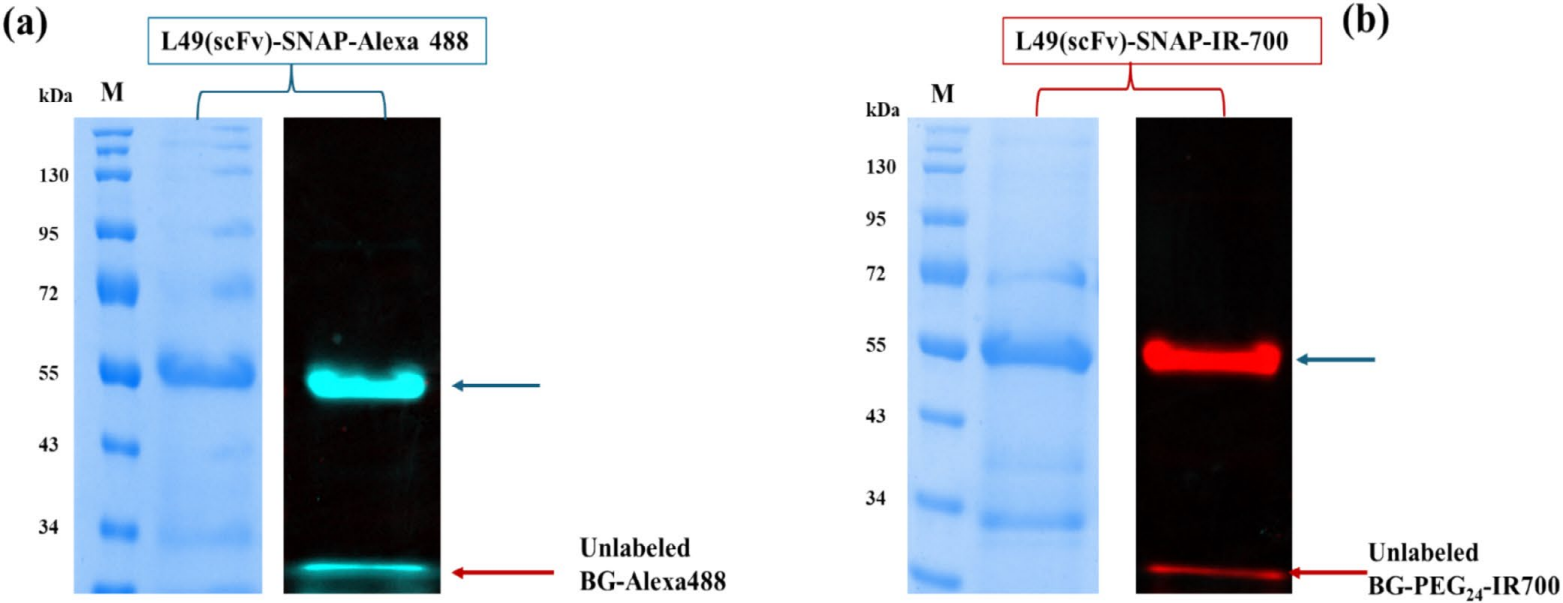
Site‐specific conjugation of the full‐length L49(scFv)‐SNAP with BG‐modified substrates. (a) Conjugation of L49(scFv)‐SNAP to BG‐Alexa 488. L49(scFv)‐SNAP was conjugated to BG‐Alexa Fluor 488 at a molar ratio of 1:2. The left‐hand side picture shows the gel stained with AcquaStain solution and the right‐hand side displays the same SDS–PAGE gel, before staining, visualized by fluorescence imaging. (b) Conjugation of L49(scFv)‐SNAP to BG‐PEG_24_‐IR700. L49(scFv)‐SNAP was incubated with a threefold molar excess of BG‐PEG_24_‐IR700 in the dark, for 3 h at room temperature to generate antibody‐photoabsorber conjugate (APC) L49(scFv)‐SNAP‐IR700. The left‐hand side picture represents the gel stained with AcquaStain solution and the right‐hand side represents the same SDS–PAGE gel, before staining, visualized by fluorescence imaging. Blue arrows indicate the successfully conjugated L49(scFv)‐SNAP and red arrows indicate the unlabeled fluorophore/dye. kDa, kilo dalton; M, protein standard; scFv, single chain variable fragment.

L49(scFv)‐SNAP was successfully conjugated to either BG‐Alexa Fluor 488 or BG‐PEG_24_‐IR700 to, respectively, generate L49(scFv)‐SNAP‐Alexa 488 or L49(scFv)‐SNAP‐IR700. Subsequently, both products, L49(scFv)‐SNAP‐Alexa 488 and L49(scFv)‐SNAP‐IR700, were run on a 10% SDS–PAGE gel and visualized through fluorescence emission using Alexa 488 (Figure 2a, right‐hand side) and IR700 (Figure 2b, right‐hand side) channels, respectively, and they appeared on the gel at the exact theoretical size of L49(scFv)‐SNAP (Figure 2). Following fluorescence acquisition, both gels were stained with AcquaStain solution, and the fusion protein L49(scFv)‐SNAP was specifically detected (left‐hand side of Figure 2a,b) at ~51.4 kDa, demonstrating C‐terminus functionality and successful self‐labeling of L49(scFv)‐SNAP.

### 3.3 Binding and Internalization of L49(scFv)‐SNAP

Binding of L49(scFv)‐SNAP can be visualized/detected after labeling to a fluorescent agent. To this end, L49(scFv)‐SNAP‐Alexa 488 was used to evaluate the binding capacity of L49(scFv)‐SNAP instead of L49(scFv)‐SNAP‐IR700 since traditional in vitro imaging devices such as confocal microscopes and flow cytometers available at UCT are unable to detect narrow or near‐infrared fluorescence peaks which correspond to the emission fluorescence wavelength of IR700 [58].

#### 3.3.1 Assessment of Cell‐Surface Binding and Internalization by Confocal Microscopy

**FIGURE 3:**
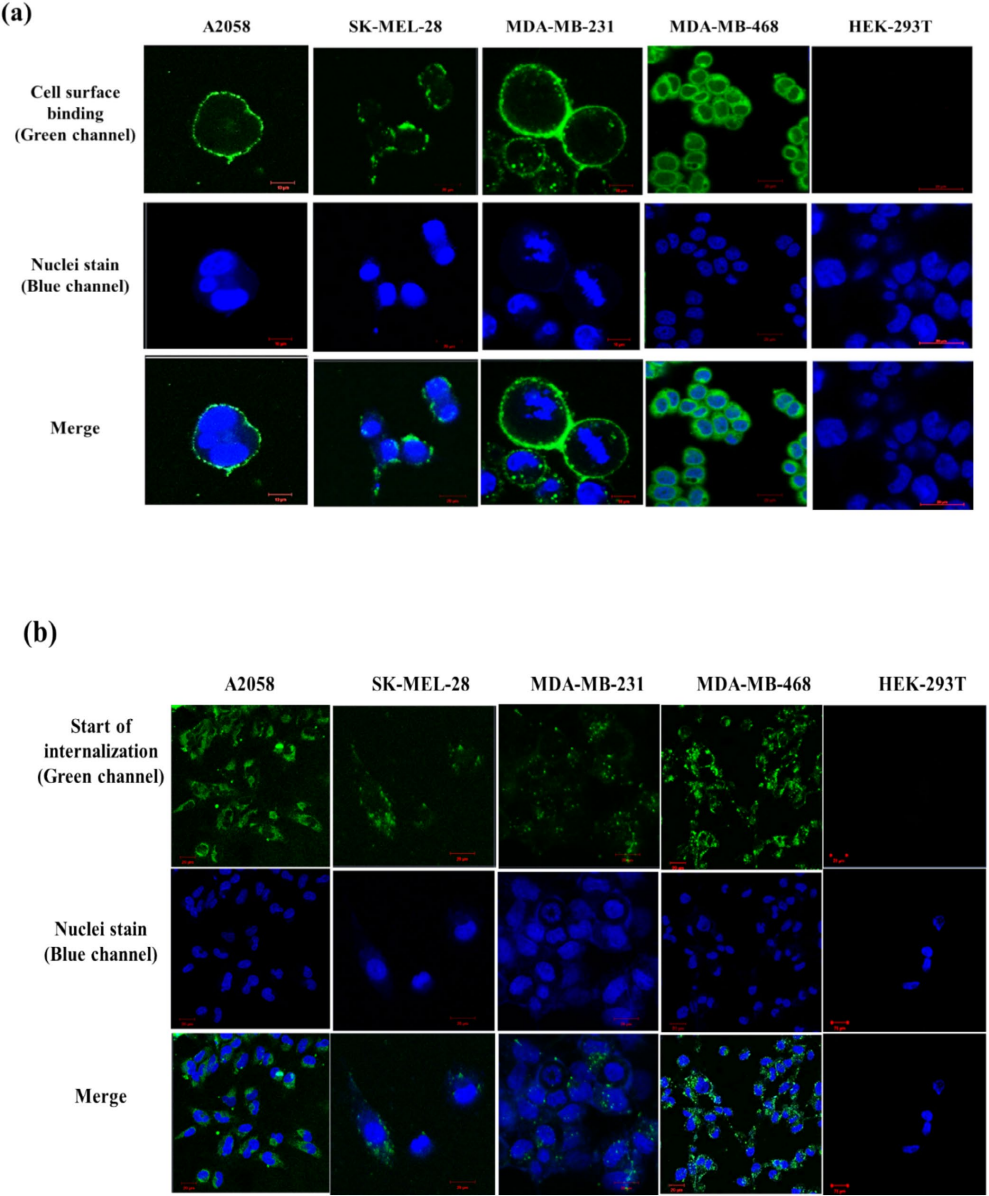
Binding and internalization studies of L49(scFv)‐SNAP‐Alexa 488. (a) Cell surface binding (live cell imaging). Surface binding L49(scFv)‐SNAP‐Alexa 488 demonstrated by the green channel was observed on melanoma and TNBC cell lines, while no binding was visible on HEK‐293T cells. (b) Internalization (fixed cell imaging). The start of internalization of L49(scFv)‐SNAP‐Alexa 488 demonstrated by the green channel was visible inside melanoma and TNBC cell lines, but not on HEK‐293T cells. Images were acquired using the Zeiss confocal scanner microscope (LSM 880, USA) with Airy Scan on the 65× and 40× air objectives, respectively, for live and fixed cell imaging. The blue channel indicates the nuclei stain by the Hoechst. Images were saved at 10 and 20 μm scale bars.

Live cell imaging of L49(scFv)‐SNAP‐Alexa 488–stained cells, carried out using confocal microscopy, revealed surface binding of L49(scFv)‐SNAP‐Alexa 488 (after 30 min) on melanoma (A2058 and SK‐MEL‐28) and TNBC (MDA‐MB‐231 and MDA‐MB‐468) cell lines, while there was no visible surface binding on HEK293T cells (Figure 3a). After 60 min incubation, L49(scFv)‐SNAP‐Alexa 488 was internalized in melanoma and TNBC cell lines, whereas no internalization was observed in HEK293T cells (Figure 3b).

#### 3.3.2 Assessment of Cell‐Surface Binding by Flow Cytometry

After confocal microscopy, flow cytometry was performed to further confirm the specific binding of L49(scFv)‐SNAP and evaluate the percentage of each cell line expressing the MTf antigen. First, the optimal concentration of L49(scFv)‐SNAP‐Alexa 488, obtained through titration steps allowing clear discrimination between MTf‐positive and negative cell lines [59, 60], was found to be 10 μg in a volume of 50 μL (0.2 μg/μL).

**FIGURE 4:**
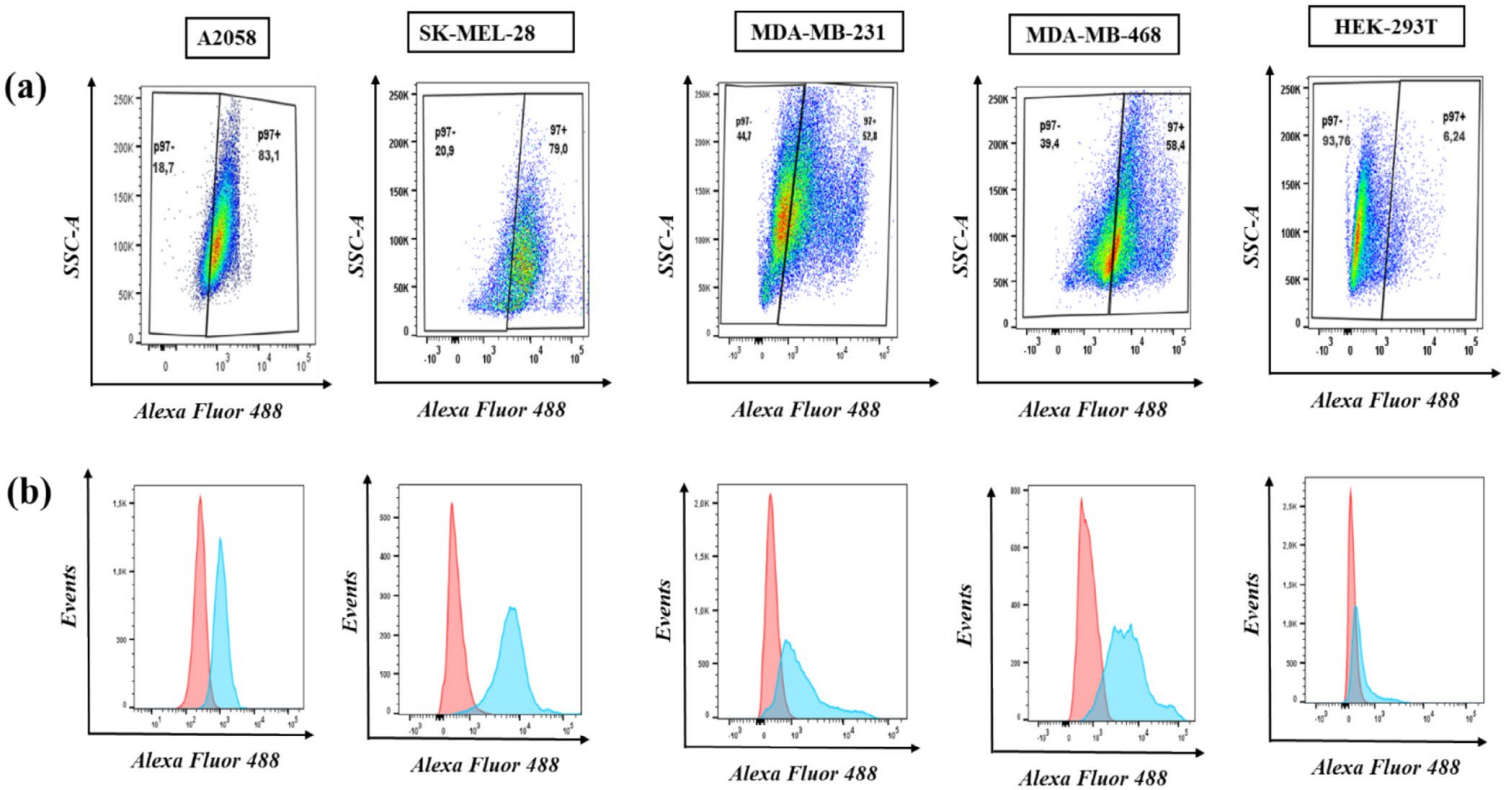
Flow cytometry measurement of L49(scFv)‐SNAP binding on melanoma and TNBC cell lines. Flow cytometry was performed following cell incubation with 10 μg of L49(scFv)‐SNAP‐Alexa 488 for 1 h on ice. (a) Pseudocolor plot's representation with gates displaying the frequency of p97(MTf)‐negative cell populations (left side of the gate) and p97(MTf)‐positive (Alexa 488+: Right side of the gate) of double stained (Live‐Dead + L49(scFv)‐SNAP‐Alexa 488) samples. (b) Histograms demonstrating the relative Alexa 488 fluorescent detection. Pink‐filled curves represent the unstained controls, while the green‐filled curves represent the double‐stained sample (Live‐Dead + L49(scFv)‐SNAP‐Alexa 488). The area where both curves are merged represents the p97‐negative cells. Data were acquired using a BD LSRFortessa Cell Analyzer and analyzed with FlowJo software (v10.8.1) (BD Biosciences, USA).

Percentages of MTf‐positive cells among the fully stained samples were as follows: 83.1, 79, 52.8, and 58.4 for A2058, SK‐MEL‐28, MDA‐MB‐231, and MDA‐MB‐468, respectively (Figure 4). Unlike the binding of L49(scFv)‐SNAP to the MTf‐negative cell line HEK‐293T, which was insignificant (6.2%).

### 3.4 Cell Viability Assay

To investigate the phototoxicity of L49(scFv)‐SNAP‐IR700 on melanoma and TNBC cell lines, following irradiation by NIR light, cell viability was assessed by the XTT‐based colorimetric assay.

**FIGURE 5:**
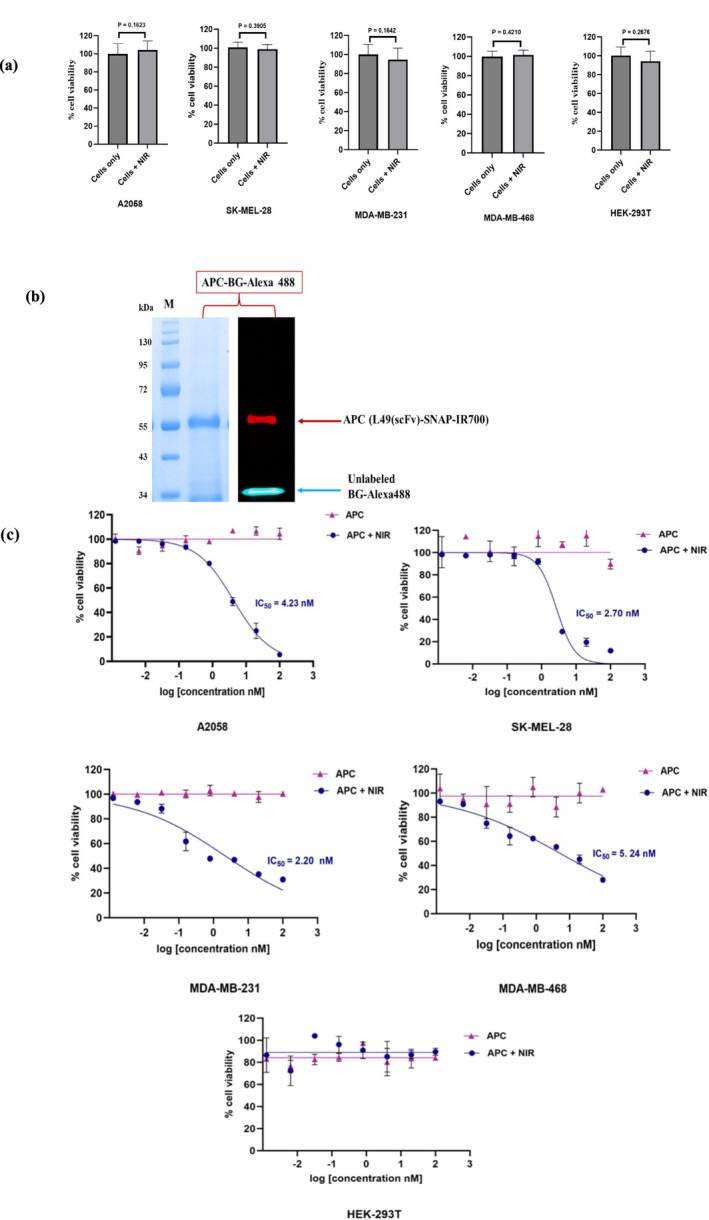
Phototoxicity of L49(scFv)‐SNAP‐IR700. (a) Effect of NIR‐light on cell lines. There was no significant difference between irradiated and non‐irradiated cells, with all P values above 0.05. (b) APC saturation. L49(scFv)‐SNAP was first incubated with a threefold molar excess of BG‐PEG_24_‐IR700 for 3 h at room temperature. Afterward, the APC was desalted and incubated with a twofold molar excess of BG‐Alexa 488. The APC (indicated with red arrow) and the unconjugated BG‐Alexa 488 (indicated with blue arrow) were visualized using the iBright FL1000 Imaging System, with both IR700 and Alexa 488 channels activated. (c) Cell viability assay of L49(scFv)‐SNAP‐IR700. Purple color curves represent cells treated with APC without irradiation and blue color curves (APC + NIR) represent cells treated with APC and irradiated with NIR light. All experiments were conducted in triplicate and repeated in three technical replicates. Data were analyzed using GraphPad Prism v10.1.1 software (https://www.graphpad.com/). APC, antibody‐photoabsorber conjugate; kDa, kilo dalton; M, protein standard; NIR, near‐infrared; scFv, single chain variable fragment.

The cytotoxicity effect of NIR‐light (690 nm) alone was first investigated. The cell viability of irradiated cells was unaffected. Likewise, there was no significant difference between irradiated and non‐irradiated cells (Figure 5a).

To ensure optimum phototoxicity during the NIR‐PIT assay, the generated APC (L49(scFv)‐SNAP‐IR700) was incubated with BG‐Alexa Fluor 488 to ensure complete saturation of the C‐terminal region (SNAP‐tag) of L49(scFv)‐SNAP. Fluorescence imaging of the SDS–PAGE gel revealed that BG‐Alexa 488 could not label the APC, demonstrating its fully saturated self‐labeling by IR‐700 (Figure 5b). Cells were treated with fivefold serial dilutions of L49(scFv)‐SNAP‐IR700 (100, 20, 4, 0.8, 0.16, 0.032, 0.0064 and 0.00128 nM) and divided into two groups (NIR‐light irradiated and non‐irradiated). Cell viability was not affected in non‐irradiated APC‐treated cells. Moreover, MTf‐negative or less‐expressing cells (HEK‐293T) treated with L49‐scFv‐SNAP‐IR700 remained unaffected regardless of NIR‐light exposure or not (Figure 5c). In contrast, a dose‐dependent reduction in cell viability was observed with all MTf‐positive cell lines treated with APC and irradiated with NIR therapeutic light. NIR‐PIT induced antiproliferative effects with IC_50_ values of 4.23, 2.70, 2.20, and 5.24 nM for A2058, SK‐MEL‐28, MDA‐MB‐231, and MDA‐MB‐468 cell lines, respectively (Figure 5c).

## 4 Discussion

This study describes the in vitro proof of concept for utilizing a SNAP‐tag–based photoimmunotherapeutic agent for the diagnostic and selective killing of MTf‐expressing melanoma and TNBC cell lines.

MTf, first reported as a melanoma antigen [13], was recently demonstrated to be highly expressed in squamous NSCLC, colorectal, pancreatic, mesothelioma, and TNBC [15, 23], with lower expression in adults' normal tissues [14]. On the other hand, MTf overexpression was reported to favor tumor proliferation [22], whereas its knockdown correlated with suppression of tumor growth and metastasis [25]. Moreover, stable transfection and subsequent expression of MTf upregulated the expression of several genes, such as the ABCB5 transporter [24]. These findings suggest that MTf does not only play a role in angiogenesis, metastasis, and chemoresistance in MTf‐positive tumors but also imply that a therapeutic regimen directly targeting MTf could inhibit its activity. Therefore, any MTf‐targeting therapy could serve as a potential strategy to enhance therapeutic efficacy by specifically delivering cytotoxic payloads to tumor cells while inhibiting key aspects of tumor development and biology.

One of the main hurdles in the ADC landscape is the heterogeneous conjugation of the antibody moiety to the toxic payload, resulting in random DARs and yielding inconsistent pharmacokinetic, efficacy, and safety profiles [50, 51]. To overcome this technical stumbling block, SNAP‐tag technology was used to conjugate L49(scFv)‐SNAP to BG‐Alexa 488 and BG‐PEG_24_‐IR700 for optical diagnosis and selective killing of MTf‐positive cells, respectively. SNAP‐tag is a modified version of the human DNA repair enzyme alkylguanine‐DNA alkyltransferase, able to specifically react with O(6)‐BG–modified substrates through an irreversible transfer of an alkyl group to a cysteine residue within its active site [52]. The unique antibody format created by fusing SNAP‐tag to a recombinant antibody fragment, therefore, provides a simple, rapid, controlled, site‐specific, and autocatalytic method for the self‐labeling of antibodies with different synthetic small effector molecules [55, 56] in a 1:1 stoichiometry [52].

L49(scFv)‐SNAP recombinant fusion protein was successfully generated, and its site‐specific conjugation with the BG‐modified fluorophore/dye was successfully demonstrated, yielding a 1:1 stoichiometry as previously reported [52, 62, 63]. BG‐Alexa 488 fluorophore was chosen over the PS IR700 dye for optical diagnostic to circumvent a technical limitation. In fact, IR‐700 has superior imaging properties for in vivo optical imaging, while traditional in vitro imaging devices such as flow cytometers and confocal microscopes are unable to detect narrow near‐infrared fluorescence peaks [58]. The optical diagnostic of MTf‐positive melanoma and TNBC cell lines was successfully carried out as confocal microscopy showed specific surface binding and internalization of the fluorophore‐labeled fusion protein on A2058, SK‐MEL‐28, MDA‐MB‐231, and MDA‐MB‐468 cells. These findings corroborate previous studies demonstrating prominent MTf expression in those cell lines [64]. Flow cytometry–based quantitative analysis of L49(scFv)‐SNAP binding towards A2058 and SK‐MEL‐28 cell lines further confirmed high and nearly similar expression levels (83% and 79%, respectively), in concordance with previously reported expression of 1.3 × 10^5^ receptors by either cell lines [64]. Noteworthy, this study is the first‐in‐kind to demonstrate surface binding and internalization of an anti‐MTf antibody fragment (L49(scFv)‐SNAP) towards TNBC cell lines MDA‐MB‐231 and MDA‐MB‐468. These observations were further confirmed by flow cytometry analysis with 52.8% and 58.4% of MDA‐MB‐231 and MDA‐MB‐468 cell lines expressing MTf antigen, respectively. These binding results of L49(scFv)‐SNAP towards those TNBC cell lines align with a previous study that reported MTf expression in TNBC tumor models [15]. Since confocal microscopy did not reveal any surface binding on HEK293T cells, the very low MTf expression measured by flow cytometry (6%) could be attributable to autofluorescence [59] or minimal MTf expression on normal tissues [14]. TNBC lacks ER, HER‐2, and PR expression [65], which further contributes to its poor diagnostic and restrains the therapeutic options [66, 67]. Thus, the pronounced binding data generated in this study indicates that MTf could be a promising target for the diagnostic of TNBC.

PIT is a photoactivable ADC developed to overcome the lack of specificity of PDT. PIT makes use of an APC (made up of a tumor‐specific antibody conjugated to a PS) and light irradiation at a specific wavelength [44]. The success of PIT highly depends on the choice of the PS. NIR‐PIT is a newly developed PIT where the tumor‐specific antibody is conjugated to the photoabsorber molecule such as dye IRDye700DX N‐hydroxysuccinimide ester (IR700) activated with therapeutic NIR light (690–700 nm wavelength) [36]. IR‐700 is a promising PS, owing to many advantageous characteristics including water solubility without biohazard properties (ensuring safety and easy excretion in urine), high purity, excellent photostability, high extinction coefficient, high therapeutic wavelength or strong absorption peak (about 690–700 nm) (which improves light penetration into tissues) and a fluorescent quantum yield higher than many other PSs (e.g., photofrin) [36, 46, 68]. The lack of passive diffusion properties is another noticeable advantage making IR700 a valuable choice of PS [36]. In addition to its therapeutic effect, fluorescence imaging of IR700 demonstrated a better detection of gastrointestinal stromal tumors in comparison to positron emission tomography (PET) or ultrasound [69]. Interestingly, the fluorescence emitted by IR700 can help track the biodistribution and tumor localization, especially during surgery procedures, offering real‐time feedback for optimizing the optimal photoirradiation timing or evaluating treatment efficacy [70].

IR700 was modified with a BG linker (BG‐PEG_24_‐NH_2_) using the NHS ester‐amino group reaction [71] without affecting its phototoxic activity [58, 72, 73]. Polyethylene glycol (PEG) is a non‐cleavable, hydrophilic and biocompatible linker here preventing the premature dissociation of IR700 from the antibody moiety and enhancing drug availability to the tumor site and therefore its therapeutic index [74]. The use of SNAP‐tag technology to generate the current APC L49(scFv)‐SNAP‐IR700 resulted in a homogeneous photoimmunotheranostic agent with a DAR of 1, ensuring highly reproducible results, unlike previous methods that randomly yielded APC with variable DARs between 0 and 6 [36, 75, 76]. In this study, generated APC was added to two groups of monolayer cells in a fivefold decrease serial concentration manner. The first group named “APC + NIR” or “NIR‐PIT” group was irradiated with NIR‐light at a therapeutic wavelength of IR700 (690–700 nM) at a dose of 40 J/cm^2^ which is the dose of the NIR‐light where Sioud et al. [61] observed the maximum cells killing in vitro [61]. In the “APC + NIR” group, a selective and dose‐dependent phototoxicity against MTf‐overexpressing cells was demonstrated with IC_50_ values in the range of nanomolar from 2.20–5.24 nM towards melanoma (A2058 and SK‐MEL‐28) and TNBC (MDA‐MB231 and MDA‐MB‐468) cells while MTf‐negative/lower expressing HEK‐239T cells were not affected. These data are about 10 times lower than previous in vitro studies on SNAP‐tag–based NIR‐PIT (with DAR of 1) targeting EGFR antigen 32–55 nM [58]. However, as discussed above, the cytotoxic effect of any ADC highly depends on the intensity of expression of the receptor on the surface of the cells [15, 77]. Thus, IC_50_ values of NIR‐PIT generated in this study further reflect the pronounced cell surface binding of the antibody moiety L49(scFv) on those observed through confocal microscopy. Additionally, the cell viability of cells irradiated with NIR‐light without APC in those in the second group treated with APC only without irradiation was not affected. Demonstrating that APC alone or NIR‐light alone has no cytotoxic effect has been demonstrated previously [58].

After APC binding to the targeted TAAs, NIR light initiates IR700 release, which leads to photochemical changes and cell death in vitro [36, 78]. It was reported that following NIR light irradiation at the therapeutic wavelength, only APCs bound to targeted TAAs were able to induce cell death without the need to be internalized [36]. This further emphasizes the advantage of NIR‐PIT in ensuring minimal off‐target effect and, most importantly, NIR‐PIT could also escape the mechanism of resistance developed by melanosomes, side population, and ABCB5 transporters on cells expressing MTf since there is no need for the probe to be internalized [36]. More specifically, NIR light activation initiates physical changes in the shape of antibody–antigen complexes which induce physical stress within the cellular membrane, and in turn increase the transmembrane water influx leading to cell bursting and necrotic cell death [78]. The subsequent release of tumor‐specific antigens (TSA) activates a rapid and highly selective immunogenic cell death (ICD) of the targeted population allowing eradication of distant metastatic cancer cells while sparing nearby cells not expressing the targeted TSA [46]. Additionally, it appears that PIT can activate a long‐lasting antitumor immune response and destroy both primary irradiated and distant non‐irradiated tumor metastases when combined with immune checkpoint blockade (ICB) therapy [79, 80, 81]. Another advantageous feature of the novel generated photoactivatable ADC generated in this study is the use of a scFv instead of a whole mAb, which allows the simple introduction of site‐specific conjugation sites, efficient penetration in solid tumors, and rapid clearance by renal filtration [58, 82].

IR700‐based immunotheranostic agents hold many therapeutic promises and have demonstrated their in vivo efficacy in several other cancer types [36, 83]. The anti‐EGFR antibody cetuximab conjugated to IR‐700 (Cetuximab sarotalocan‐RM‐1929/ASP‐1929) was conditionally approved (September 2020) for the treatment of unresectable locally advanced or recurrent HNSCC (jRCT2031200133) and fully approved for the treatment of recurrent head and neck squamous cell carcinoma (rHNSCC) in Japan [84]. Since then, this probe has been approved in other regions and is currently under Phase 3 clinical trial (NCT03769506) in patients with inoperable rHNSCC in Asia, America, and EU [46, 85, 86].

## 5 Conclusion

This study provides a framework for the use of a scFv fused to SNAP‐tag, allowing optical diagnostic and selective killing of cancer cell lines expressing melanoma/p97 antigen in vitro. The findings further highlight the necessity of preclinical in vivo studies of SNAP‐tag–based APCs for NIR‐PIT, since they could be an effective therapeutic regimen for light‐accessible melanotransferrin‐positive melanoma and TNBC. Engaging this novel APC in efficacy and pharmacokinetic/toxicity investigations using mouse models is warranted to further validate the current proof‐of‐concept study.

## Author Contributions

Conceptualization: S.H.M. and S.B. Methodology: S.H.M., F.A.N.B., G.T.S., and S.B. Investigation: S.H.M. Results discussion: S.H.M. and S.B. Software: S.H.M. and G.T.S. Validation: S.H.M. and S.B. Resources: D.L. and S.B. Writing – original draft preparation: S.H.M. Writing – review and editing: S.H.M., G.T.S., F.A.N.B., N.L., and S.B. Supervision, project administration, and funding acquisition: S.B.

## Ethics Statement

The authors have nothing to report.

## Conflicts of Interest

The authors declare no conflicts of interest.

## Data Availability

Relevant data supporting the findings of this study are available within this article.
